# Disturbed hippocampal intra-network in first-episode of drug-naïve major depressive disorder

**DOI:** 10.1093/braincomms/fcac323

**Published:** 2022-12-08

**Authors:** Keita Watanabe, Naomichi Okamoto, Issei Ueda, Hirofumi Tesen, Rintaro Fujii, Atsuko Ikenouchi, Reiji Yoshimura, Shingo Kakeda

**Affiliations:** Open Innovation Institute, Kyoto University, Kyoto 6068501, Japan; Department of Psychiatry, University of Occupational and Environmental Health, Kitakyushu 8078555, Japan; Department of Radiology, Graduate School of Medicine, Hirosaki University, Hirosaki 0368502, Japan; Department of Psychiatry, University of Occupational and Environmental Health, Kitakyushu 8078555, Japan; Department of Psychiatry, University of Occupational and Environmental Health, Kitakyushu 8078555, Japan; Department of Psychiatry, University of Occupational and Environmental Health, Kitakyushu 8078555, Japan; Department of Psychiatry, University of Occupational and Environmental Health, Kitakyushu 8078555, Japan; Department of Radiology, Graduate School of Medicine, Hirosaki University, Hirosaki 0368502, Japan

**Keywords:** hippocampal network, structural covariance network, hippocampus, major depression

## Abstract

Complex networks inside the hippocampus could provide new insights into hippocampal abnormalities in various psychiatric disorders and dementia. However, evaluating intra-networks in the hippocampus using MRI is challenging. Here, we employed a high spatial resolution of conventional structural imaging and independent component analysis to investigate intra-networks structural covariance in the hippocampus. We extracted the intra-networks based on the intrinsic connectivity of each 0.9 mm isotropic voxel to every other voxel using a data-driven approach. With a total volume of 3 cc, the hippocampus contains 4115 voxels for a 0.9 mm isotropic voxel size or 375 voxels for a 2 mm isotropic voxel of high-resolution functional or diffusion tensor imaging. Therefore, the novel method presented in the current study could evaluate the hippocampal intra-networks in detail. Furthermore, we investigated the abnormality of the intra-networks in major depressive disorders. A total of 77 patients with first-episode drug-naïve major depressive disorder and 79 healthy subjects were recruited. The independent component analysis extracted seven intra-networks from hippocampal structural images, which were divided into four bilateral networks and three networks along the longitudinal axis. A significant difference was observed in the bilateral hippocampal tail network between patients with major depressive disorder and healthy subjects. In the logistic regression analysis, two bilateral networks were significant predictors of major depressive disorder, with an accuracy of 78.1%. In conclusion, we present a novel method for evaluating intra-networks in the hippocampus. One advantage of this method is that a detailed network can be estimated using conventional structural imaging. In addition, we found novel bilateral networks in the hippocampus that were disturbed in patients with major depressive disorders, and these bilateral networks could predict major depressive disorders.

## Teaser

Using a novel method to describe hippocampal intra-network based on structural imaging of MRI imaging, Watanabe *et al*. report a disturbed network that works bilateral hippocampal tail in major depressive disorders. Further, the intra-hippocampal network might be used as a diagnostic aid for major depressive disorders.

## Graphical Abstract

**Graphical abstract:**
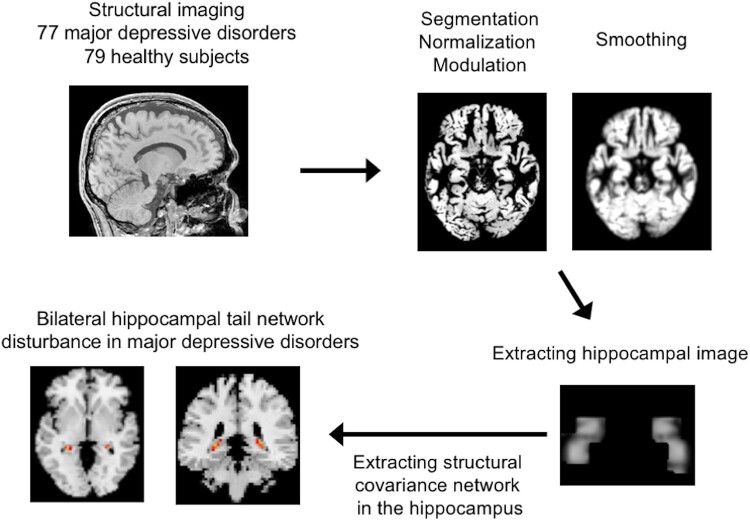

## Introduction

The hippocampus is thought to play an important role in major depressive disorder. Previous meta-analyses have revealed a reduction in hippocampal volume in major depressive disorder.^1-3^ Furthermore, reduced hippocampal volume may be associated with the risk of major depressive disorder^4^ and the severity of depressive symptoms.^5^

In terms of network topology, the hippocampus has been characterized as a hub, exhibiting structural covariance with multiple other brain regions in keeping with its central role in multiple cognitive processes.^6^ In contrast, there exists a complex network inside the hippocampus.^7^ To evaluate intra-networks, previous studies used resting-state functional MRI and hippocampal subfield segmentation techniques.^8,9^ However, there are highly discrepant segmentation protocols for hippocampal subfield segmentation.^10^ In addition, even with a high-resolution setting, the resolution of resting-state functional MRI may not support hippocampal subfield segmentation.^11,12^ Therefore, it is not easy to depict the intra-networks in the hippocampus from resting-state functional MRI.

**Figure 1:**
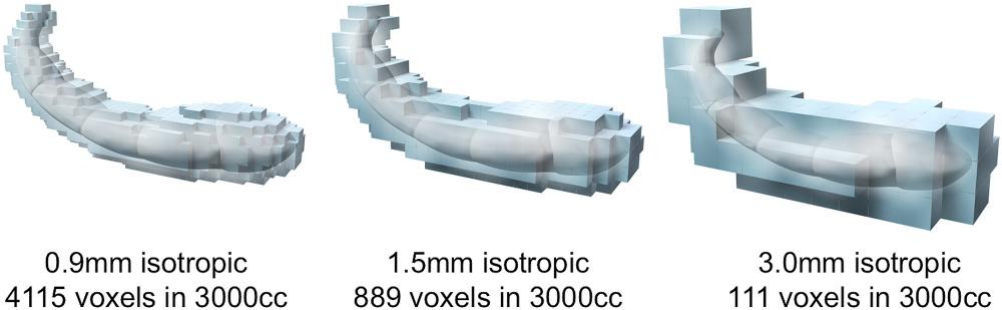
**Voxel size and hippocampus.** The images illustrate the relationship between the hippocampus and isotropic voxel sizes; 0.9 mm: the current study size, 1.5 mm: conventional VBM analysis size converted from original structural imaging, 3.0 mm: conventional functional MRI size.

To evaluate the network across the brain using MRI, structural imaging was used as the structural covariance network, in addition to functional MRI and diffusion tensor imaging. Structural covariance networks arise due to neural plasticity; regions that fire and wire together might also be coupled in particular volumes because of mutually trophic and plasticity-related changes at the synaptic and cellular levels.^13^ Although not identical, the structural covariance networks are thought to allow the evaluation of brain networks in a way similar to resting-state functional MRI such as default mode network^14^ and basal ganglia network.^15^ Compared with the grey matter (GM) volume analysis using voxel-based morphometry (VBM), the structural covariance network analysis can improve sensitivity to detect the GM differences in psychiatric disorders.^16^ Furthermore, in multi-site structural imaging analysis, the structural covariance network can reduce noise and scanner effects.^17^ In addition, structural imaging has the advantage of a high spatial resolution, which is considered important for evaluating the hippocampus. For instance, with a total volume of 3 cc, the hippocampus contains 3000 voxels for a 1 mm isotropic voxel size of conventional structural imaging or ∼111 voxels for a 3 mm isotropic voxel of conventional functional or diffusion tensor imaging (Fig. 1). Therefore, we hypothesized that information on the detailed intra-network of the hippocampus could be extracted by utilizing the high spatial resolution of structural imaging. The structural images of patients with psychiatric disorders have been accumulated in numerous studies and evaluating the intra-network of the hippocampus from structural imaging may greatly advance the understanding of brain abnormalities in psychiatric disorders.

To evaluate the intra-network in the hippocampus, we combined the advantages of high spatial resolution of conventional structural imaging and structural covariance networks. We aimed to extract the intra-network in the hippocampus using the high spatial resolution of conventional original structural imaging and investigate intra-network abnormalities in patients with major depressive disorders.

## Materials and methods

### Participants

All human experiments were performed in accordance with the guidelines provided and approved by the Institutional Review Board of the University of Occupational and Environmental Health School of Medicine, Japan (approval number: H25-13). All participants provided written informed consent to participate in this study.

This study included 77 first-episode drug-naïve patients with major depressive disorder and 79 healthy subjects (HSs). All subjects had participated in our previous study^12^ that had analysed the whole-brain structural network using conventional source-based morphometry (SBM). A well-trained psychiatrist diagnosed patients with major depressive disorders using a fully structured clinical interview for diagnosis and statistical manual for mental disorders, fourth edition text revision (DSM-IV-TR) (SCID) between March 2009 and July 2018. Furthermore, patients with major depressive disorders were required not to have previously met the criteria for any DSM-IV-TR Axis I disorder. In addition, only patients with a 17-item Hamilton Rating Scale for Depression (HAMD-17) score ≥14, mild to moderate or higher severity,^18^ were eligible for inclusion in the study.

The psychiatrist excluded patients with a history of neurological disease or the presence of either Axis I (schizophrenia, other affective disorders, and so on) or Axis II (personality disorders, mental retardation, and so on) psychiatric disorders. In total, 79 HSs were recruited from nearby communities through an interview conducted by the same psychiatrist using the full SCID-I, non-patient edition. None of the HSs had a history of serious medical or neuropsychiatric illnesses or a family history of major psychiatric or neurological illnesses among their first-degree relatives. Detailed information is described in a previous study.^12^

### MRI acquisition

A 3T MRI system (Signa EXCITE 3T; GE Healthcare, Waukesha, WI, USA) was used for MR images of three-dimensional fast-spoiled gradient-recalled acquisition. An eight-channel brain phased-array coil was equipped. We set the acquisition parameters as follows: repetition time, 10 ms; echo time, 4.1 ms; inversion time, 700 ms; flip angle, 10°; matrix, 256 × 256; field-of-view, voxel size, 0.9 × 0.9 × 1.2 mm. After zero-interpolation filling, the recon parameters were as follows: recon matrix 512 × 512; recon voxel size, 0.47 × 0.47 × 0.6 mm. We corrected image distortions and intensity inhomogeneity using the Grad Warp software program^19^ and ‘N3’ function,^20^ respectively. MRI parameter is also described in a previous study.^12^

### Image processing

The process of conventional VBM analyses with SPM12 software (Statistical Parametric Mapping 12; Institute of Neurology, London, UK)^21,22^ was used for pre-processing of images. The structural images in native space were spatially normalized, segmented into GM, white matter and cerebrospinal fluid images, and modulated using the Diffeomorphic Anatomical Registration Through Exponential Lie Algebra (DARTEL) toolbox in SPM12.^23^ DARTEL is considered as an accurate method for normalization.^21^ In contrast to conventional VBM processing, to maintain high spatial resolution, the voxel size was set at 0.9 mm isotropic voxel size, which is normally converted to a 1.5 mm isotropic voxel size.

**Figure 2:**
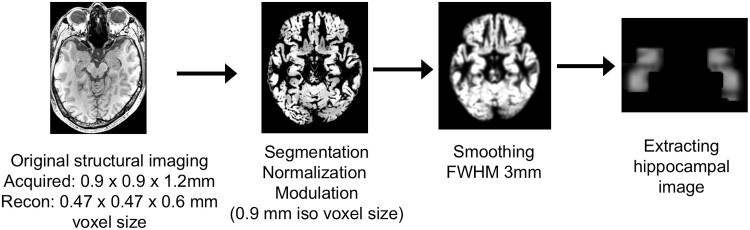
**Image processing flow.** The image processing applied Conventional VBM analysis using SPM 12. Voxels of the hippocampus were extracted. To maintain high-resolution, the voxel size was set as 0.9 mm isotropic in the normalization process, and a 3 mm full width at half maximum Gaussian kernel was used in the smoothing process.

Furthermore, the resulting modulated GM images were smoothed using a 3 mm full width at half maximum Gaussian kernel. After the smoothing process, we extracted the hippocampal image defined by automated anatomical labelling^24^ using the WFU PickAtlas version 3.0.4.^25,26^  Figure 2 shows the process flow.

To identify structural networks among hippocampal voxels, SBM analysis was performed using the group independent component analysis (ICA) of functional MRI toolbox (http://icatb.sourceforge.net).^16^ The minimum description length principle suggested nine reliable independent components (ICs). We performed ICA using a neural network algorithm (Infomax) that attempts to minimize the mutual information of the network outputs to identify naturally grouping and maximally independent sources.^27^ ICA was repeated 20 times to investigate the reliability of ICA estimates of ICASSO^28^ to ensure the stability of the estimated components as in earlier works.^29-32^ We selected the mode of RandInit. The min and max cluster size was set as 16 and 20, respectively.

As a result, a matrix showed the 156 rows of 156 subjects (79 HS and 77 major depressive disorder). Columns indicate a voxel. ICA decomposed the matrix into two matrices. The first matrix comprises a subject per row and an IC per column. This matrix involves loading coefficients that show how each IC contributes to the 156 subjects. Therefore, this matrix includes data on the relationship between each subject and each IC. The loading coefficients reflect the expression of specific brain structural covariance networks for each subject. The second matrix specifies the relationship between the ICs and the voxels.

To visualize the ICs, the source matrix was reshaped back to a three-dimensional image, scaled to unit standard deviations (*Z* maps), and the threshold at |*z*| > 3.0.

### Statistical analysis

Statistical analyses were conducted using Statistical Package for the Social Sciences (SPSS; version 27.0, IBM Corp., Armonk, NY, USA). Differences were considered statistically significant at *P* < 0.05. In the analysis of multivariate analysis of covariance (MANCOVA) and binominal logistic regression analysis, a threshold of <0.007 using Bonferroni correction was applied to find components showing a significant effect of diagnosis from seven components.

To compare the demographic characteristics between patients with major depressive disorder and HSs, a two-tailed *t*-test was performed to assess differences in age and years of education. The *χ*^2^ test was used for sex comparisons. In the MANCOVA analysis, the diagnosis group (HS and major depressive disorder) was entered as an independent variable, while all loading coefficients were calculated to indicate intra-network connectivity in the hippocampus. Age, sex and years of education were used as covariates. In addition, binominal logistic regression analysis was used to assess whether the intra-networks in the hippocampus could predict major depressive disorder. To confirm whether major depressive disorder can be predicted from only MRI information, only the loading coefficients of networks were used as predictive variables, and age and gender were not used. A receiver operating characteristic (ROC) curve analysis was also performed based on the prediction formula of the logistic regression analysis.Results

### Baseline demographic data

Table 1 shows the baseline demographic data of the participants. There were significant differences in age and years of education between HSs and major depressive disorders, but there were no significant differences between the sexes.

**Table 1:** Demographic data

	MDD (*n* = 77)	HS (*n* = 79)	*P* value
Age, mean (range, SD)	52.0 (22–73, 15.1)	38.9 (20–65, 10.2)	<0.01
Female, numbers	44	34	0.08
Years of education, mean (SD)	13.4 (2.5)	16.7 (3.0)	<0.01
HAMD-17, mean of total scores (SD)	22.6 (5.9)		

HAMD-17 = 17-item Hamilton Rating Scale for Depression; MDD = major depressive disorders; SD = standard deviation.

### Intra-network in hippocampus

**Figure 3:**
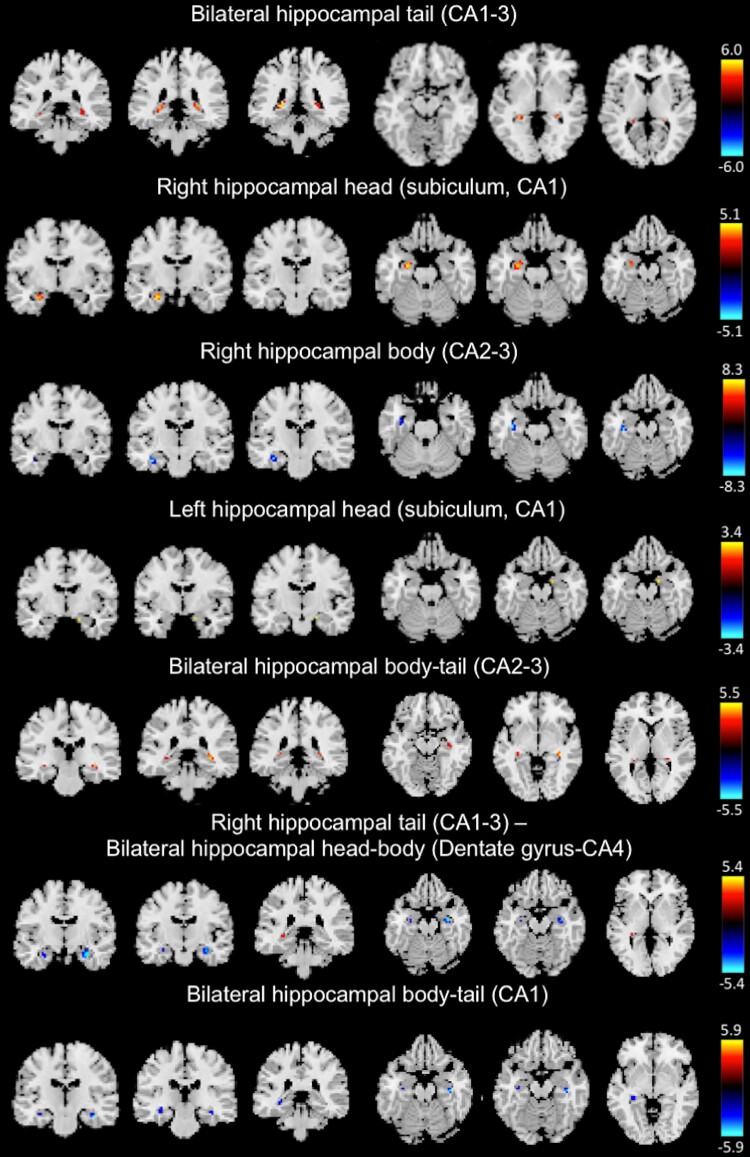
**Voxel-based structural covariance intra-network in the hippocampus.** The figures show the structural covariance networks with |*Z*| > 3.0. Red/yellow: voxel volumes show a positive correlation. Blue/light blue: the voxel volumes show a negative correlation.

The ICA analysis generated nine ICs. Two experienced neuroradiologists reviewed these components. The anatomical regions of the hippocampal subfields were determined based on an illustrated tutorial research paper^33^ with the consent of neuroradiologists. Two of these components were determined to be artefacts based on the criteria defined by Xu *et al*.:^16^ components containing several sharp edges near the boundary of the brain or those appearing primarily in regions that do not contain GM. These components were excluded from the subsequent analyses. Figure 3 and Table 2 show the ICs representing the structural covariance networks in the hippocampus. The two characteristics were as follows: bilateral networks [bilateral hippocampal tail (cornu ammonis: CA1–3), bilateral hippocampal body–tail (CA2–3), right hippocampal tail (CA1–3), bilateral hippocampal head–body (dentate gyrus-CA4), bilateral hippocampal body–tail (CA1)] and networks along the longitudinal axis [bilateral hippocampal tail (CA1–3), right hippocampal body (CA2–3) and bilateral hippocampal body–tail (CA2–3)].

**Table 2:** Intra-networks in hippocampus

Anatomical regions of structural covariance network	Volume (cc) left/right	Max *z*-value for left/right hemisphere (Talairach coordinates *x*, *y*, *z*)
Bilateral hippocampal tail (CA1–3)		
	0.3/0.4	7.3 (−25, −38, 2)/5.8 (28, −36, 2)
	0.3/0.1	6.0 (−22, −38, 5)/5.2 (25, −36, 5)
Right hippocampal head (subiculum, CA1)		
	0.6/0.0	5.6 (−26, −10, −22)/0 (0, 0, 0)
	0.2/0.0	4.4 (−23, −9, −22)/0 (0, 0, 0)
	0.3/0.0	3.1 (−23, −6, −19)/0 (0, 0, 0)
	0.2/0.1	3.2 (−36, −27, −12)/2.6 (26, −45, 4)
Right hippocampal body (CA2–3)		
	0.8/0.0	9.4 (−37, −17, −18)/0 (0, 0, 0)
	0.4/0.1	8.2 (−36, −14, −20)/2.8 (34, −8, −25)
Left hippocampal head (subiculum, CA1)		
	0.1/0.6	2.7 (−20, −16, −14)/3.5 (20, −15, −15)
	0.0/0.3	0 (0, 0, 0)/2.8 (20, −2, −21)
	0.1/0.0	2.9 (−26, −10, −23)/0 (0, 0, 0)
Bilateral hippocampal body–tail (CA2–3)		
	0.3/0.7	4.4 (−29, −35, 2)/5.6 (33, −32, −1)
	0.2/0.1	3.3 (−33, −27, −6)/4.2 (29, −33, 4)
	0.0/0.2	0 (0, 0, 0)/2.8 (37, −25, −14)
Right hippocampal tail (CA1–3)-bilateral hippocampal head–body (dentate gyrus-CA4)		
	
	
	0.3/0.0	3.4 (−30, −34, 0)/0 (0, 0, 0)
	0.1/0.3	3.1 (−30, −11, −19)/5.6 (32, −11, −20)
	0.1/0.6	3.4 (−26, −10, −23)/5.5 (27, −9, −23)
Bilateral hippocampal body–tail (CA1)		
	0.2/0.0	3.4 (−36, −36, −9)/0 (0, 0, 0)
	0.1/0.4	3.3 (−35, −20, −15)/6.4 (38, −18, −18)
	0.4/0.3	4.3 (−33, −30, −7)/6.2 (37, −14, −19)

Table presents max *z*-value and Talairach coordinate of areas with a volume of 0.1 cc or more.

### Intra-network abnormality in major depression

The correlations among age and loading coefficients of seven intra-networks in HS and major depressive disorder were shown in the correlation matrix (Supplementary Fig. 1). Table 3 shows the results of MANCOVA. There was a significant difference in the bilateral hippocampal tail (CA1–3) network (*P* = 0.004, *ηp*^2^ = 0.05).

**Table 3:** Differences between MDD and HS using a MANCOVA analysis

	*F*	*ηp* ^2^	*P*
Bilateral hippocampal tail (CA1–3)	8.53	0.05	0.004*
Right hippocampal head (subiculum, CA1)	1.36	0.01	0.25
Right hippocampal body (CA2–3)	1.62	0.01	0.21
Left hippocampal head (CA1)	0.20	0.00	0.66
Bilateral hippocampal body–tail (CA2–3)	0.31	0.00	0.58
Right hippocampal tail (CA1–3)— Bilateral hippocampal head–body (dentate gyrus-CA4)	1.31	0.01	0.25
Bilateral hippocampal body–tail (CA1)	2.61	0.02	0.11

Age, sex and years of education were set as covariates. An asterisk indicates significance after Bonferroni correction.

**Figure 4:**
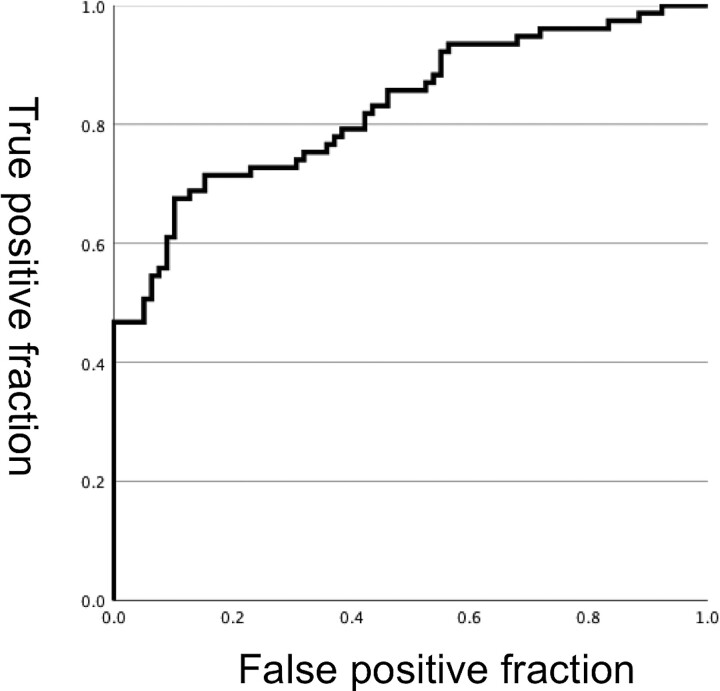
**ROC curve.** Receiver operating characteristic curves for performance of logistic regression to predict major depressive patients using the intra-networks in the hippocampus. The area under the curve was 0.83. When the cut-off value was set to 0.5 with the equation obtained from the regression analysis, the sensitivity, specificity and accuracy were 84.6, 71.4 and 78.1%, respectively.

In a binomial logistic regression analysis, bilateral networks, including the bilateral hippocampal tail (CA1–3) and bilateral hippocampal body–tail (CA2–3) were significant predictors for major depressive disorder (*P* = 0.00002, 0.001, respectively) (Table 4). When the cut-off value was set to 0.5 with the equation obtained from the regression analysis, the sensitivity, specificity and accuracy were 84.6, 71.4 and 78.1%, respectively. In the ROC analysis, the area under the curve was 0.83 (Fig. 4).

**Table 4:** Binominal logistic regression model for predicting MDD

	OR	Lower CI	Upper CI	*P*
Bilateral hippocampal tail (CA1–3)	3.62	2.01	6.52	0.00002*
Right hippocampal head (subiculum, CA1)	0.53	0.27	1.02	0.06
Right hippocampal body (CA2–3)	1.38	0.90	2.13	0.14
Left hippocampal head (CA1)	1.15	0.59	2.26	0.68
Bilateral hippocampal body–tail (CA2–3)	0.30	0.15	0.60	0.001*
Right hippocampal tail (CA1–3)— Bilateral hippocampal head–body (dentate gyrus-CA4)	0.57	0.35	0.92	0.02
Bilateral hippocampal body–tail (CA1)	1.90	1.14	3.14	0.01

An asterisk indicates significance after Bonferroni correction.

CI = confidence interval; OR = odds ratio.

## Discussion

The examination of the intrinsic connectivity of each voxel in the hippocampus revealed two major results. First, multiple networks connecting the bilateral hippocampi were described, not only in the ipsilateral hippocampus. Second, major depressive disorder showed a significant difference in the networks connecting the bilateral hippocampal tails (CA1–3). In addition, the bilateral hippocampal networks are predictors of major depressive disorder. To the best of our knowledge, this is the first study to assess intra-network abnormalities in the hippocampi of patients with major depressive disorder.

### Intra-network in the hippocampus using MRI

While many studies have investigated non-uniform structural changes using hippocampal subfield segmentation, MRI studies of the intra-network in the hippocampus are still developing. In the HSs, the intra-network was described using resting-state functional images of ∼1.5 mm isotropic^34-36^ and 3.0 mm isotropic^37^ voxels on 3T MRI and 1.5–2.0 mm isotropic voxels on 7T MRI^8,38^ combined with hippocampal subfields segmentation from structural imaging. In these previous studies, functional networks of bilateral^8^ and along the longitudinal axis^38^ were found, consistent with the structural covariance networks in this study. Regarding intra-network alterations in the hippocampus, age-related dysfunction in the network along the longitudinal axis was observed.^36,39^ Furthermore, the network along the longitudinal axis may contribute to memory decline.^36^

In this study, we extracted the intra-networks in the hippocampus based on SBM and ICA. SBM and ICA consider information across different voxels and extract naturally occurring covariance patterns. This data-driven method examines the connectivity of each voxel without prior identification of the hippocampal subfields. In addition, a previous study on cross-modal integration focused on the structural covariance and resting-state functional network revealed that structural covariance networks are more likely to reflect short-distance connections.^40^ Because the intra-network in the hippocampus contains very short-distance connections, such as synapse connections, the method presented in this study is considered suitable for evaluating the intra-network in the hippocampus.

### Major depression disorder and intra-network in the hippocampus

Accumulating evidence indicates that major depressive disorder shows structural and functional changes in the hippocampus^41,42^ and large-scale network dysfunction across the brain on resting-state functional MRI.^43,44^ Regarding structural changes in the hippocampus, subfield-specific changes are considered to exist.^45-47^ However, findings on subfield-specific changes in major depressive disorders are inconsistent because they may depend not only on medication use^48^ and illness duration^49^ but also on the diversity of segmentation protocols.^10^ Regarding hippocampal network abnormality in major depressive disorder, recent studies have focused on the connectivity of specific hippocampal subfields with the rest of the brain.^50,51^ For instance, it has been reported that a disturbed functional network between the right anterior hippocampus and insula correlates with the symptoms of depression in major depressive disorder.^50^

Furthermore, bilateral intra-networks in the hippocampus have been shown to predict major depressive disorder. In this study, we found disturbed bilateral hippocampal tail (CA1-3) networks in patients with major depressive disorder. However, there were no significant differences in the networks along the longitudinal axis. Although recent findings have highlighted the network along the longitudinal axis as a key contributor to the functional organization,^52,53^ the underlying function of bilateral networks remains unclear. Interestingly, a recent study separated the structural and functional networks into two models: (i) bilateral hippocampal formations and (ii) bilateral extra-hippocampal structures by mapping networks of the medial temporal lobe using graph theory and hippocampal subfield segmentation on 7T MRI.^8^ Thus, both the ICA based on each voxel and graph theory based on hippocampal subfields revealed bilateral networks. Future research on the bilateral hippocampal networks found by new analysis methods is needed to reveal their role in brain function and psychiatric disorders. The finding of lateralized morphometric changes of the hippocampus in major depressive disorder^54,55^ may be related to the bilateral network in the hippocampus.

### Study limitations

A limitation of our study is that functional MRI was not performed to confirm the intra-network in the hippocampus extracted from the structural MRI. However, a previous study showed a direct association between functional networks and structural covariance networks using SBM across the entire brain.^15^ Furthermore, in the intra-network in the hippocampus based on hippocampal subfield segmentation, a correlation between structural and functional networks has been reported.^8^

## Conclusion

We present a novel method for evaluating voxel-based structural covariance networks within the hippocampus. One advantage of this method is that a detailed network can be estimated using conventional structural imaging. In addition, we found novel networks in the bilateral hippocampus that were disturbed in patients with major depressive disorder. Furthermore, the bilateral networks in the hippocampus could predict major depressive disorders (MDD).

## Supplementary Material

fcac323_Supplementary_Data

## Data Availability

The data that support the findings of this study are available from the corresponding author, upon reasonable request.

## Abbreviations

CA =: cornu ammonis
DARTEL =: diffeomorphic anatomical registration through exponential Lie Algebra
DSM-IV-TR =: diagnosis and statistical manual for mental disorders, fourth edition text revision
GM =: grey matter
HAMD-17 =: 17-item Hamilton Rating Scale for Depression
HSs =: healthy subjects
IC =: independent component
ICA =: independent component analysis
MANCOVA =: multivariate analysis of covariance
ROC =: receiver operating characteristic
SBM =: source-based morphometry
SCID =: structured clinical interview for DSM-IV-TR
SPSS =: Statistical Package for the Social Sciences
VBM =: voxel-based morphometry
