# Supplementary material for: Disturbed hippocampal intra-network in first-episode of drug-naïve major depressive disorder
Source: Brain Commun. 2022 Dec 8;5(1):fcac323. doi: 10.1093/braincomms/fcac323 (PMC9798279; doi:10.1093/braincomms/fcac323)
Supplement: fcac323_Supplementary_Data [file fcac323_supplementary_data.pdf]

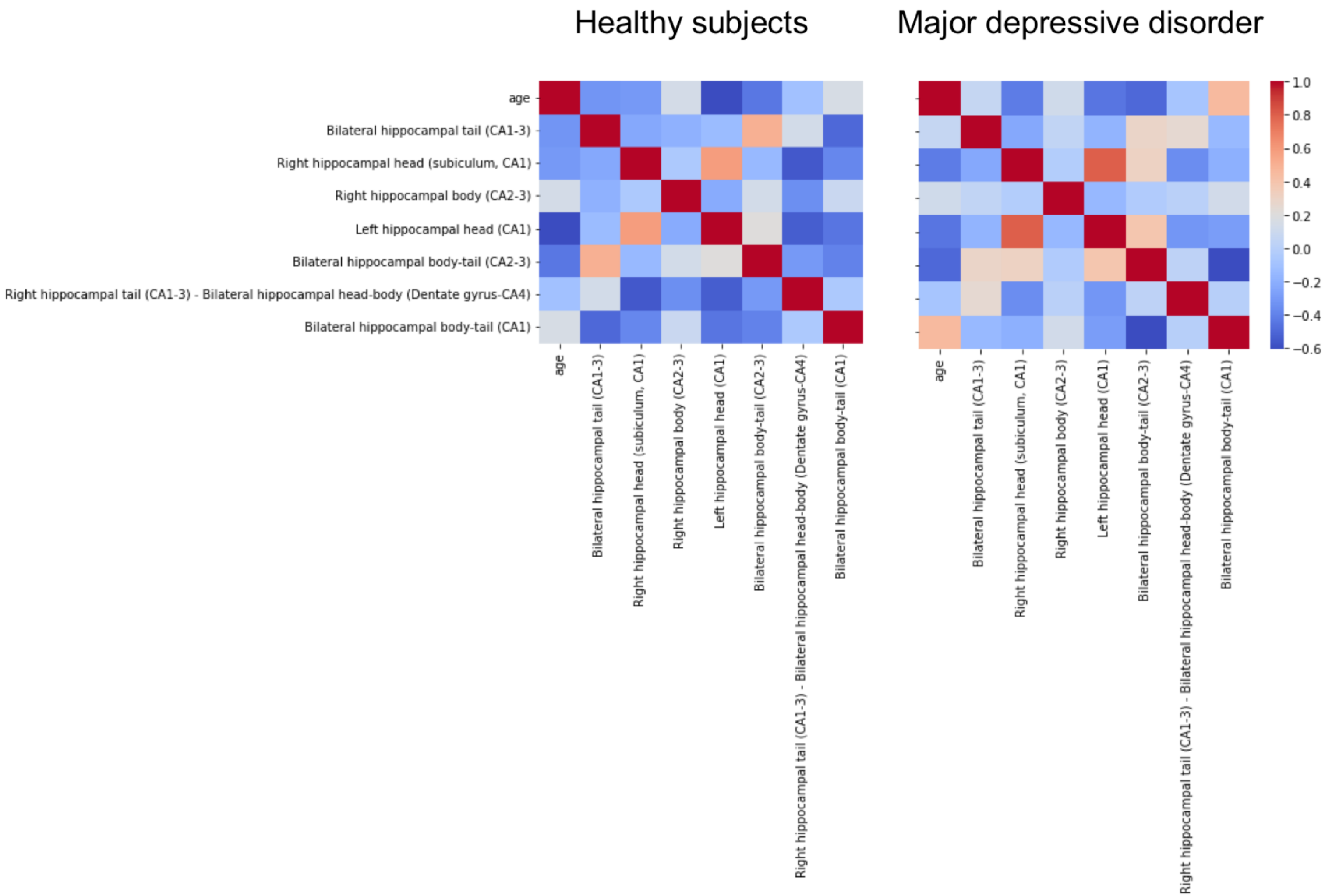

Supplemental Figure 1 Correlation matrix

The correlation matrix shows the correlation coefficients among age and loading coefficients of 7 intra-networks in the hippocampus. An asterisk shows the significance of $p < 0.05$ based on two tailed Pearson correlation test.
